# Functional Interdependence of Anoctamins May Influence Conclusions from Overexpression Studies

**DOI:** 10.3390/ijms25189998

**Published:** 2024-09-17

**Authors:** Jiraporn Ousingsawat, Rainer Schreiber, Karl Kunzelmann

**Affiliations:** Physiological Institute, University of Regensburg, University Street 31, D-93053 Regensburg, Germany; jiraporn.ousingsawat@vkl.uni-regensburg.de (J.O.); rainer.schreiber@ur.de (R.S.)

**Keywords:** anoctamin 6, ANO6, TMEM16F, phospholipid scrambling, Ca^2+^ signaling, ion currents

## Abstract

Anoctamin 6 (ANO6, TMEM16F) is a phospholipid (PL) scramblase that moves PLs between both plasma membrane (PM) leaflets and operates as an ion channel. It plays a role in development and is essential for hemostasis, bone mineralization and immune defense. However, ANO6 has also been shown to regulate cellular Ca^2+^ signaling and PM compartments, thereby controlling the expression of ion channels such as CFTR. Given these pleiotropic effects, we investigated the functional interdependence of the ubiquitous ANO6 with other commonly co-expressed anoctamins. As most expression studies on anoctamins use HEK293 human embryonic kidney cells, we compared ion currents, PL scrambling and Ca^2+^ signals induced by the overexpression of anoctamins in HEK293 wild-type parental and ANO6-knockout cells. The data suggest that the endogenous expression of ANO6 significantly affects the results obtained from overexpressed anoctamins, particularly after increasing intracellular Ca^2+^. Thus, a significant interdependence of anoctamins may influence the interpretation of data obtained from the functional analysis of overexpressed anoctamins.

## 1. Introduction

Anoctamin 6 (ANO6, TMEM16F) is a PL scramblase that allows the transport of PLs between both plasma membrane (PM) leaflets. It also operates as a nonselective ion channel that is permeable for Ca^2+^ ions [1,2,3]. ANO6 is expressed in every cell type examined so far. In this regard, it is an essential protein that fulfills basic cellular tasks, apart from cell-specific functions during development, hemostasis, bone mineralization and immune defense [4]. It has also been shown to regulate intracellular Ca^2+^ ([Ca^2+^]_i_) signaling and exocytosis and thus it controls large PM reservoirs that determine the PM expression of ion channels such as CFTR [5,6,7,8,9]. Apart from ANO6, nine additional members belong to the family of anoctamins, with ANO1 and ANO2 operating as Ca^2+^-activated Cl^-^ channels in acini of exocrine glands, smooth muscle cells, sensory neurons, and brain cells, respectively [10,11,12,13,14,15]. While ANO8 tethers the endoplasmic reticulum (ER) and controls [Ca^2+^]_i_ signals [16,17], it does not seem to conduct ions or scramble membrane PLs. The other seven members are PL scramblases and are often (but not exclusively) located intracellularly [18,19].

Given the pleiotropic effects reported for ANO6 and the other nine members of the anoctamin family [19], we wondered whether the expression of ANO6 itself also affects the function of other anoctamins when overexpressed in HEK293 cells. By shaping [Ca^2+^]_i_ signals, membrane scrambling, and controlling intracellular vesicular transport/exocytosis, endogenous ANO6 can exert cellular effects that were originally attributed to overexpressed (and endogenous) anoctamins [19]. The goal of the present study was therefore to compare the cellular effects induced by overexpressed anoctamins in wild-type HEK293 cells expressing endogenous ANO6 (parental) with HEK293 cells lacking expression of ANO6 (KO-ANO6). In addition, we compared these results with acute siRNA-based knockout in HEK293T cells, which stably express the SV40 large T-antigen, thus binding SV40 enhancers of expression vectors to increase the production of proteins such as CFTR [20].

We chose HEK293 and HEK293T cells as they express significant levels of endogenous ANO6 [13] and because they are frequently used in overexpression studies [3,21,22,23,24]. We compared ion transport, PL scrambling and [Ca^2+^]_i_ signals related to the expression of different anoctamins in parental and KO-ANO6 cells. We detected remarkable differences regarding the activation of ion currents, PL scrambling, and the anoctamin-dependent regulation of [Ca^2+^]_i_ signaling, suggesting a significant influence of ANO6 on the results obtained in studies with overexpressed anoctamins. 

## 2. Results

### 2.1. Complete Knockout of ANO6 Versus Acute Knockdown of ANO6: Differences in [Ca^2+^]_i_ Signaling and Activation of CFTR 

**Figure 1:**
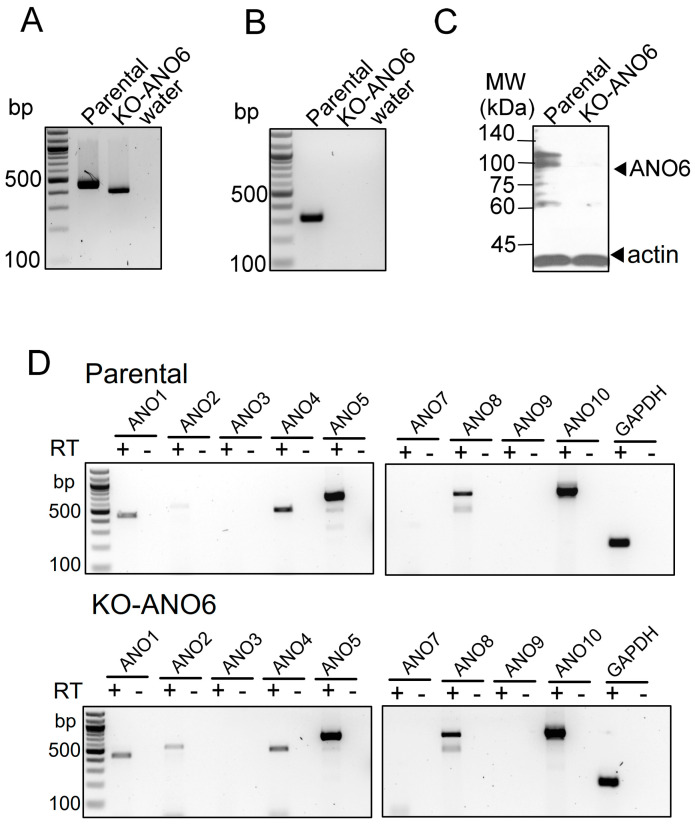
Knockout of ANO6 in HEK293 cells. (**A**) RT-PCR of ANO6 expressed in HEK293 parental and HEK293 KO-ANO6 cells. Reduced band size in KO-ANO6 cells indicates deletion in exon 5. (**B**) RT-PCR with a 3′ primer downstream of the deletion did not produce a band in KO-ANO6 cells due to the premature stop codon. (**C**) Western blotting of ANO6 in parental cells and lack of ANO6 expression in KO-ANO6 cells. (**D**) RT-PCR showing comparison of expression of other anoctamins in parental cells and HEK293 KO-ANO6 cells. All blots and RT-PCR were performed as triplicates.

The expression of ANO6 was knocked out in HEK293 cells (KO-ANO6) applying CRISPR-Cas9 technology to HEK293 parental cells, introducing a stop codon at position 167 (Ubigene Biosciences, Austin, USA). RT-PCR analysis demonstrated the reduced size of the amplified mRNA fragment in KO-ANO6 cells (Figure 1A). RT-PCR with a 3′ primer downstream of the deletion did not produce any band in KO-ANO6 cells (Figure 1B). Western blotting of endogenous ANO6 amplified in parental and KO-ANO6 cells indicated a lack of expression of ANO6 in KO-ANO6 cells (Figure 1C). We compared the endogenous expression of other anoctamins in parental and KO-ANO6 cells by RT-PCR and detected a band for ANO2 and the expression of ANO4, ANO5, ANO8, and ANO10 in both parental and KO-ANO6 cells (Figure 1D). 

**Figure 2:**
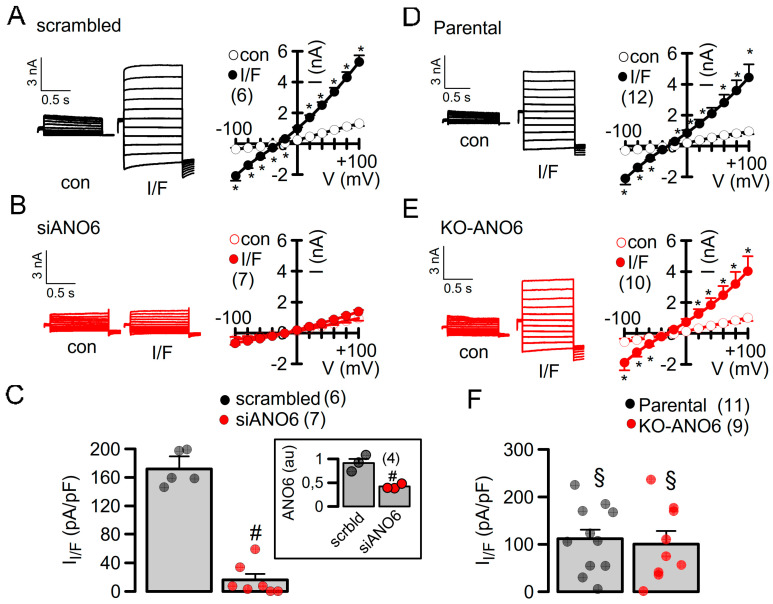
CFTR is not activated after siRNA-knockdown of ANO6 but can be stimulated in KO-ANO6 cells. (**A**,**B**) Whole-cell current overlays and current/voltage (I/V) relationships of CFTR-expressing HEK293 cells treated with scrambled RNA (*p* = 0.00011) (**A**) or siRNA for ANO6 (no activation) (**B**). Cells were stimulated with IBMX (100 µM) and forskolin (2 µM) to activate CFTR. (**C**) Summary of current densities for I/F-activated whole-cell currents indicates abolished CFTR currents in cells treated with siRNA for ANO6. Inset indicates significant inhibition of ANO6 expression by siRNA (*p* = 0.0013) au = arbitrary units. (**D**,**E**) Comparison of CFTR whole-cell currents in parental (*p* = 0.00092) and KO-ANO6 (*p* = 0.0011) cells. (**F**) Summary of current densities indicates attenuated CFTR currents, which, however, were present in both parental and KO-ANO6 cells (*p* = 0.0018 and *p* = 0.0021). Mean ± SEM (number of experiments). * significant activation by I/F (*p* < 0.05; paired *t*-test). ^#^ significant difference when compared to scrambled (*p* < 0.05; unpaired *t*-test). ^§^ significant difference when compared to scrambled (*p* < 0.05; unpaired *t*-test).

Previously, we demonstrated the role of ANO1 and ANO6 for membrane expression and the activation of CFTR in the airways and intestines of animals with a tissue-specific knockdown of ANO1 expression, as well as various cell lines [9,25]. Here, we examined the role of ANO6 in the activation of CFTR by overexpressing CFTR in parental (wt) HEK293 cells with cells in which ANO6 was knocked down or knocked out. Thus, CFTR was expressed in HEK293T cells treated with siRNA-ANO6 or scrambled RNA, or was measured in HEK293 parental cells and KO-ANO6 cells. Endogenous ANO1 was downregulated by siRNA in all cells in order to avoid contaminating ANO1 currents [25]. The acute siRNA-knockdown of ANO6 in HEK293T cells completely eliminated the activation of CFTR by IBMX and forskolin, fully confirming previous results (Figure 2A–C) [9]. However, quite surprisingly, we still found activation of CFTR in KO-ANO6 cells (Figure 2D–F).

**Figure 3:**
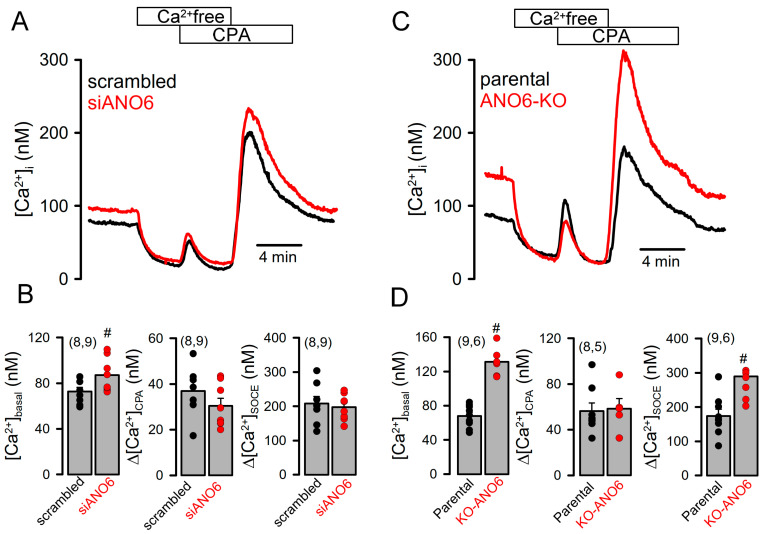
Knockout of ANO6 enhances basal Ca^2+^ influx and store-operated Ca^2+^ entry (SOCE). (**A**,**C**) Measurement of [Ca^2+^]_i_ using Fura2. Removal of extracellular Ca^2+^ (Ca^2+^ free) and subsequent emptying of ER Ca^2+^ stores by cyclopiazonic acid (CPA; 10 µM). Re-addition of extracellular Ca^2+^ in the presence of CPA induced a rise in intracellular Ca^2+^. (**B**,**D**) Summaries for the effects of siRNA-knockdown of ANO6 expression and ANO6-knockout on basal Ca^2+^ (*p* = 0.029 and *p* = 0.0078), CPA-induced rise in [Ca^2+^]_i_ (no differences) and SOCE (not significant and *p* = 0.0092). Mean ± SEM (number of experiments). ^#^ significant difference between siRNA and scrambled or parental and KO-ANO6 (*p* < 0.05; unpaired *t*-test).

In KO-ANO6 cells, CFTR currents could still be activated, which prompted us to examine [Ca^2+^]_i_ levels in these cells, as earlier studies showed that [Ca^2+^]_i_ is essential for the activation of CFTR [26,27]. We found that [Ca^2+^]_i_ signals were highly upregulated in KO-ANO6 cells—[Ca^2+^]_i_ was largely enhanced due to an enhanced basal Ca^2+^ influx and augmented store-operated Ca^2+^ entry (SOCE). This was demonstrated by the emptying of intracellular ER Ca^2+^ stores through the application of cyclopiazonic acid (CPA), an inhibitor of the endoplasmic Ca^2+^ uptake pump, SERCA (Figure 3A,C). The reasons for the pronounced increase in SOCE appear to be related to the loss of ANO6 expression, as similar changes were observed with siRNA-ANO6 knockdown in HEK293T cells, which, however, did not reach significance (Figure 3A–D).

### 2.2. The Function of Anoctamins Is Influenced by ANO6

**Figure 4:**
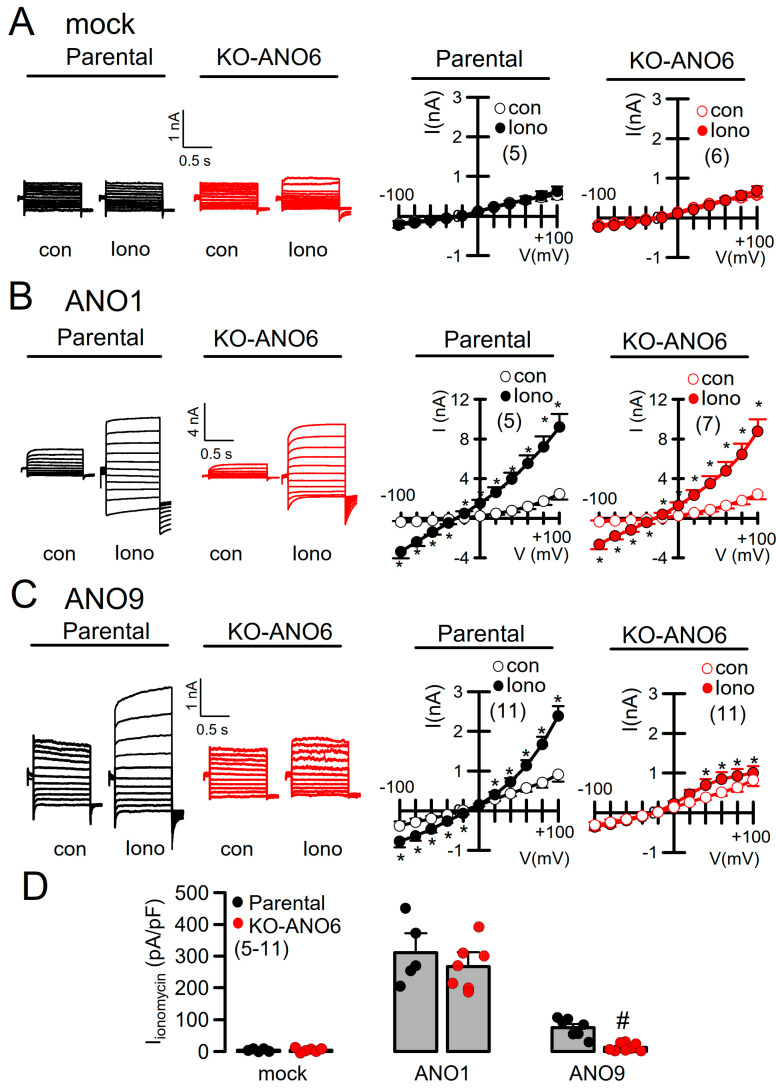
Effects of ANO6 knockdown on whole-cell currents by overexpressed ANO1 and ANO9. Whole-cell current overlays and I/V curves in parental and KO-ANO6 cells stimulated with Iono (1 µM) in mock transfected cells (no activation) (**A**) and cells overexpressing ANO1 (*p* = 0.0032 and *p* = 0.0041) (**B**) or ANO9 (*p* = 0.0034 and *p* = 0.041) (**C**). (**D**) Summary for Iono-induced current densities detected in parental and KO-ANO6 cells (*p* = 0.0048). Mean ± SEM (number of experiments). * significant activation by Iono (*p* < 0.05; paired *t*-test). ^#^ significant difference between siRNA and scrambled or parental and KO-ANO6 (*p* < 0.05; unpaired *t*-test).

Although [Ca^2+^]_i_ was strongly upregulated in KO-ANO6 cells, no whole-cell currents were activated under basal conditions or after stimulation with the Ca^2+^ ionophore ionomycin (Iono) in mock-transfected control cells (Iono; Figure 4A). As we and other laboratories made extensive use of HEK293 cells to study the properties of the Ca^2+^-activated Cl^-^ channel ANO1, the scramblases ANO3, -4, -5, -9 and -10, and the tether protein ANO8, we examined the impact of endogenous ANO6 on the cellular functions induced by overexpressed anoctamins. ANO1 overexpressed in KO-ANO6 cells could be readily activated and was only slightly smaller when compared to ANO1 currents activated in parental cells (Figure 4B). Thus, ANO1 expression and function appear largely independent of ANO6. ANO9 whole-cell currents were detected previously [18,22,28]. Notably, the activation of ANO9 by Iono, as shown in parental cells, was almost abolished in KO-ANO6 cells (Figure 4C,D). Thus, ANO9 may require ANO6 to enhance its membrane expression, or ANO9 may facilitate the membrane expression of ANO6, as both ANO6 and ANO9 are PL scramblases and produce ion currents. We therefore continued by examining the potential role of ANO6 for PL scrambling induced by other anoctamins (ANO3, -4, -5, -8, -9 and -10), which are mainly (but not exclusively) expressed in intracellular compartments [13].

**Figure 5:**
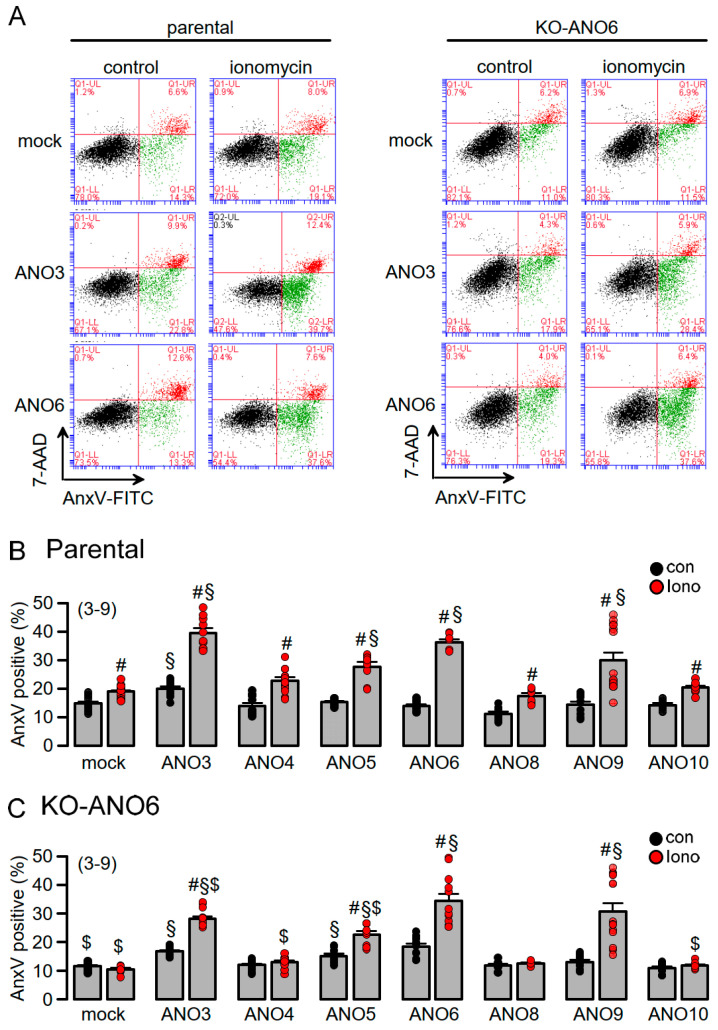
PL scrambling by intracellular anoctamins depends on expression of ANO6. (**A**) Four-quadrant blots for parental and KO-ANO6 cells expressing ANO3 or ANO6 or mock-transfected cells under control conditions and after stimulation with Iono (10 µM/10 min). Summary of Iono-induced Annexin V exposure (% cells positive for AnxV) in parental (**B**) and KO-ANO6 cells (**C**). Cells overexpressed various, predominantly intracellular, anoctamins or were mock-transfected. Mean ± SEM (number of experiments). # significant activation by Iono (*p* = 0.038–0.0087; unpaired *t*-test). ^§^ significant difference when compared to mock (*p* = 0.044–0.018; ANOVA). ^$^ significant difference when compared to parental (*p* = 0.047–0.0088; unpaired *t*-test).

PL scrambling by intracellular anoctamins depends on the expression of ANO6: The fraction of annexin V (AnxV)-positive cells indicating the exposure of phosphatidylserine was assessed by flow cytometry. In parental cells, 16 % of mock-transfected control cells were AnxV-positive, and this fraction was further enhanced by stimulation with Iono and was not detectable in KO-ANO6 cells (Figure 5A–C). Basal scrambling (no Iono) was slightly enhanced by the expression of ANO3, while cells overexpressing ANO3, -5, -6 and -9 demonstrated enhanced Iono-activated scrambling. Iono-activated scrambling was attenuated in ANO3- and -5- but not ANO9-expressing cells. ANO4, -8 and -10 did not induce PL scrambling in KO-ANO6. Taken together, endogenous ANO6 adds scrambling activity but otherwise does not seem to regulate scramblase activity by overexpressed scramblases. The results obtained in KO-ANO6 cells demonstrate the lack of scramblase activity of ANO4, -8 and -10.

### 2.3. The Effects of Anoctamins on [Ca^2+^]_i_ Signals Depends on Expression of ANO6

**Figure 6:**
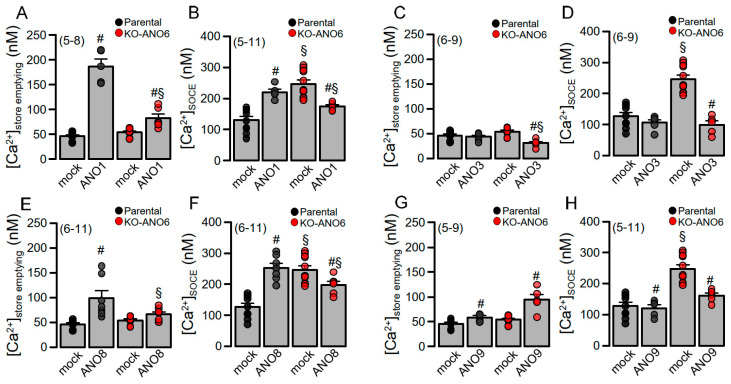
The effects of anoctamins on [Ca^2+^]_i_ signals depend on the expression of ANO6. Summaries for CPA/Ca^2+^-free induced ER-store emptying (**A**,**C**,**E**,**G**) (*p* = 0.042–0.0091) and SOCE (**B**,**D**,**F**,**H**) (*p* = 0.045–0.0017) in parental and KO-ANO6 cells expressing ANO1, ANO3, ANO8, and ANO9. Mean ± SEM (number of experiments). ^#^ significant difference when compared to mock (*p* < 0.05; unpaired *t*-test). ^§^ significant difference when compared to parental (*p* < 0.05; unpaired *t*-test).

Anoctamins were shown to affect [Ca^2+^]_i_ signals [11,28,29,30]. We therefore looked into the impact of ANO6 on anoctamin-modulated Ca^2+^ signals using a so-called ER Ca^2+^-store-emptying protocol (c.f. Methods). The PM-localized Ca^2+^-activated Cl^−^ channel ANO1 tethers the ER to the PM [5,11] and thus strongly augmented CPA-induced store emptying and store-operated Ca^2+^ entry (SOCE) (Figure 6A,B). ANO1-enhanced store emptying was strongly attenuated in KO-ANO6 cells, while enhanced SOCE in KO-ANO6 cells was surprisingly inhibited by ANO1. In contrast, the expression of ANO3 had no effects on Ca^2+^ signals but inhibited store emptying and SOCE in the absence of ANO6 (Figure 6C,D). The membrane tether ANO8 [16] enhanced store emptying and SOCE, both being inhibited in KO-ANO6 cells (Figure 6E,F). Finally, ANO9 alone did not affect Ca^2+^ signals in parental cells but enhanced store emptying and inhibited SOCE in the absence of ANO6 (Figure 6G,H). In summary, ANO6 modulates the expression of ion channels and changes in Ca^2+^ signaling caused by the expression of other anoctamins.

## 3. Discussion

When overexpressed, anoctamins exert pleiotropic cellular effects due to their functions as ion channels, PL scramblases and membrane tethers [19]. Soon after their initial discovery, it was noticed that mammalian cells express variable numbers (2-8) of anoctamin paralogues [13] (Figure 1C). The expression of these anoctamins is not constant but highly variable, depending on differentiation, proliferation, cell cycles, and other factors. For example, apical ANO1, i.e., Ca^2+^ activated Cl^-^ currents, are hardly detectable in fully differentiated cells of the airways and intestine, but they are strongly expressed after isolation and during proliferation [31]. In turn, the differential expression of anoctamins such as ANO6 may affect transcription or PM expression of other anoctamins and ion channels such as CFTR or Orai1 (Figure 1, Figure 3 and Figure S1) [9]. Moreover, ion currents attributed to the expression of ANO5, ANO9, or ANO10 were dependent on the presence (ANO9) or absence (ANO5 and -10) of ANO6 (Figure S1C). This result could be explained by a differential regulation of the PM expression of these anoctamins.

ANO6 is expressed in any cell type and has a pronounced impact on PM turnover (unfolding, exo- and endocytosis) due to its property as a PL scramblase [5,6,7,8,9,32]. ANO6 may operate as a housekeeper protein that accomplishes basic cellular tasks, apart from cell-specific functions [33,34,35,36]. Because of its intracellular/PM localization, ANO6 may regulate intracellular vesicle traffic and exocytosis, thereby affecting the PM expression of other proteins, as suggested in previous reports [6,32,37]. In preliminary biotinylation experiments, we detected an enhanced PM expression of the Ca^2+^ influx channel Orai1 in KO-ANO6 cells, while Orai1-mRNA was not upregulated. This may explain the augmented SOCE in KO-ANO6 cells (Figure S1A,B). Overall, endogenous ANO6 provides a small scramblase activity in addition to the PL scramblase induced by ANO3 and ANO5, but not by ANO9 (Figure 5). ANO6 PL scrambling, exocytosis and membrane shedding all drive PM expression of proteins, potentially altering the entire extracellularly exposed proteome, thus leading to pleiotropic cellular effects induced by ANO6 [6,32,38,39]. Finally, the expression of ANO1 enhanced scrambling likely by facilitating the Ca^2+^-dependent activation of ANO6, as previously reported [40].

Attenuated Ca^2+^ influx has been reported for ANO3 and ANO9, which are both intracellular and PM-localized scramblases [28,30]. For some anoctamins examined here, their effects on Ca^2+^ signaling was dependent on the expression of ANO6 (Figure 6). ANO6 is also required for the PM expression of CFTR channels [9], which, surprisingly, was only detected in HEK293T cells. HEK293T cells are derived from HEK293 cells but stably express the SV40 large T-antigen, which can bind to SV40 enhancers of expression vectors to increase protein production. These cells proliferate more rapidly and express higher levels of CFTR, which, however, also reduces the stability of CFTR [20]. These properties could explain why the ANO6 dependence of CFTR PM expression was detected in HEK293T cells but not in HEK293 cells. However, the high [Ca^2+^]_i_ levels present in HEK293 KO-ANO6 supports membrane expression and the activation of CFTR, as previously reported [9,25,26,41,42] (Figure 3).

## 4. Materials and Methods

### 4.1. Cell Culture and Transfection

HEK293T (Sigma, Deisenhofen, Germany), HEK293 parental and HEK293-ANO6 knockout cell lines (KO-ANO6; Ubigene Biosciences, Austin, TX, USA) were cultured in DMEM (Dulbecco’s Modified Eagle Medium), supplemented with 10% FBS Xtra and 1% penicillin (100,000 units/liter)/streptomycin (100 mg/liter) (Capricorn Scientific, Ebsdorfergrund, Germany). All cells were cultured at 37 °C in a humidified atmosphere of 5% (v/v) CO_2_. Cells were transfected with plasmid vectors or siRNA using standard protocols for Lipofectamine 3000 (Thermo Fisher Scientific, Waltham, MA, USA). All experiments were performed 48 h after transfection. 

### 4.2. RT-PCR

For semi-quantitative RT-PCR, total RNA from HEK293 WT and HEK293 KO-ANO6 cells were isolated using NucleoSpin RNA II columns (Macherey-Nagel, Düren, Germany). Total RNA (0.5 µg / 25 µL reaction) was reverse-transcribed using random primers (Promega, Mannheim, Germany) and M-MLV Reverse Transcriptase RNase H Minus (Promega, Mannheim, Germany). Each RT-PCR reaction contained sense and antisense primers (both 0.5 µM) (Table 1), 0.5 µL cDNA and GoTaq Polymerase (Promega, Mannheim, Germany). After 2 min at 95 °C, cDNA was amplified (targets 35 cycles, reference GAPDH 25 cycles) for 30 s at 95 °C, 30 s at 56 °C and 1 min at 72 °C. PCR products were visualized by loading on Midori Green Xtra (Nippon Genetics Europe, Düren, Germany) containing agarose gels and were analyzed using Image J 1.52r (NIH, Bethesda, MD, USA).

### 4.3. Validation of Deletion in Exon5 of ANO6

The deletion of 59 bp (Ubigene Biosciences, Austin, TX, USA) caused a frame shift and a stop codon at position 167. The deletion was validated by the company, and we further confirmed the correct sequence by PCR (WT: 463 bp, KO-ANO6: 404 bp, primer Table 1) and sequencing (Microsynth AG, Balgach, Switzerland; primer Table 1). 

### 4.4. Measurement of [Ca^2+^]_i_ Concentrations

Cells were grown on coated glass coverslips and loaded with 2 μM Fura-2, AM (BIOZOL, Holland, OH, USA) with 0.02% Pluronic F-127 (Invitrogen, Waltham, MA, USA) in ringer solution (in mM: NaCl 145, KH_2_PO_4_ 0.4, K_2_HPO_4_ 1.6, Glucose 5, MgCl_2_ 1, Ca- Gluconate 1.3) for 2 h at room temperature. Fluorescence was detected in cells perfused with Ringer’s solution at 37 °C using an inverted microscope (Axiovert S100, Zeiss, Oberkochen, Germany) and a high-speed polychromator system (VisiChrome, Puchheim, Germany). Fura-2 was excited at 340/380 nm, and the emission was recorded between 470 and 550 nm using a CCD-camera (CoolSnap HQ, Visitron Systems, Puchheim, Germany). The control of the experiment, imaging acquisition, and data analysis were conducted with the software package Meta-Fluor version 4 (Universal imaging, Miami Lakes, FL, USA). [Ca^2+^]_i_ was calculated from the 340/380 nm fluorescence ratio after background subtraction. The formula used to calculate [Ca^2+^]_i_ was [Ca^2+^]_i_ =Kd x (R-R_min_)/(R_max_-R) x (S_f2_/S_b2_), where R is the observed fluorescence ratio. The values R_max_ and R_min_ (maximum and minimum ratios) and the constant S_f2_/S_b2_ (fluorescence of free and Ca^2+^-bound Fura-2 at 380 nm) were calculated using 2 µM Iono (Cayman Chemical, Biomol GmbH, Hamburg, Germany) and 5 mM EGTA to equilibrate intracellular and extracellular Ca^2+^ in intact Fura-2-loaded cells. The dissociation constant for the 24 Fura-2•Ca^2+^ complex was taken as 224 nM. The control of the experiment, imaging acquisition, and data analysis were performed with the software package Meta-Fluor (Universal imaging, Miami Lakes, FL, USA). An ER store-emptying protocol was applied, which includes the removal of extracellular Ca^2+^ (0 Ca^2+^; detection of basal Ca^2+^ influx), the application of cyclopiazonic acid (CPA; 10 µM; a reversible blocker of the SERCA Ca^2+^ pump to empty the ER Ca^2+^ store), and the re-addition of extracellular Ca^2+^ (1.3 mM) to demonstrate the magnitude of the (ER) store-operated Ca^2+^ entry (influx; SOCE).

### 4.5. Patch Clamp

Cells were grown on coated glass coverslips. Coverslips were mounted in a perfused bath chamber on the stage of an inverted microscope (IM35, Zeiss) and kept at 37 °C. Patch pipettes were filled with a cytosolic-like solution containing (in mM) KCl 30, K- Gluconate 95, NaH_2_PO_4_ 1.2, Na_2_HPO_4_ 4.8, EGTA 1, Ca- Gluconate 0.758, MgCl_2_ 1.03, D- Glucose 5, and ATP 3 at pH 7.2. The intracellular Ca^2+^ activity was 0.1 μM. The expression of endogenous ANO1 was suppressed by siRNA in all experiments, as demonstrated by semiquantitative RT-PCR and densitometric analysis: 0.95 ± 0.06 (scrambled RNA; *n* = 3) vs. 0.35 ± 0.02 (siRNA-ANO1; n = 3). The bath was perfused continuously with standard bicarbonate-free Ringer’s solution at a rate of 4 mL/min. Patch pipettes had an input resistance of 3–5 MΩ, and whole-cell currents were corrected for serial resistance. Currents were recorded using a patch clamp amplifier, EPC9, and PULSE software (HEKA, Stuttgart, Germany, available from https://resources.pulsesoftware.com/, last accessed date 4 August 2024), as well as Chart software version 8.1.30 (AD Instruments, Sydney, Australia). Cells were stimulated with 1 μM Iono (Cayman Chemical, Biomol GmbH Germany), 100 µM 3-isobutyl-1-methylxanthine (Sigma, Deisenhofen, Germany) and 2 µM forskolin (Tocris Bioscience, Bio-Techne, Germany). In regular intervals, membrane voltage (Vc) was clamped in steps of 20 mV from −100 to +100 mV from a holding voltage of 0 mV. The current density was calculated by dividing whole-cell currents by cell capacitance.

### 4.6. Flow Cytometry

Cells were collected using accutase (Capricorn Scientific, Germany), washed with cold Dulbecco’s PBS (DPBS), and centrifuged at 500 g and 4 °C for 10 min. Cells were incubated for 10 min in 100 μL annexin binding buffer containing 5 μL annexin V-FITC and 2.5 μL 7-aminoactinomycin D (7-AAD; BioLegend, San Diego, CA, USA) with 10 Iono, DMSO, as a control. Reactions were stopped by adding 400 μL of DPBS, and cells were immediately analyzed using a BD Accuri™ C6 flow cytometer. The total events collected were at least 20,000 events per sample. 

### 4.7. Western Blotting

Protein was isolated from cells with RIPA-Buffer (Cell Signaling, Danvers, MA, USA) containing 20 mM Tris-HCl pH 7.5, 150 mM NaCl, 1mM Na_2_EDTA, 1 mM EGTA, 1% NP-40, 1% sodium deoxycholate, 2.5 mM sodium pyrophosphate, 1 mM β-glycerophosphate, 1 mM Na_3_VO_4_, and 1 µg/mL leupeptin. Protease inhibitor cocktail 1 mM PMSF (Roche Diagnostics, Rotkreuz, Switzerland) was added freshly before use. Proteins were then separated by 8.5% SDS-PAGE and transferred to a PVDF membrane (GE Healthcare, Chicago, IL, USA). Membranes were incubated overnight at 4 °C with rabbit anti-human ANO6 antibody (PA5-69345, Thermo Fisher Scientific, Waltham, MA, USA). Rabbit anti-actin (Sigma, Deisenhofen, Germany) was used as a loading control. Membranes were then incubated with horseradish peroxidase (HRP)-conjugated goat anti-rabbit secondary antibodies at room temperature for 1.5 h, and immunoreactive signals were visualized using a SuperSignal HRP chemiluminescence substrate-detection kit (Thermo Fisher Scientific, Waltham, MA, USA). 

### 4.8. Biotinylation

Cells were washed twice with ice-cold PBS (Ca^2+^/Mg^2+^), followed by incubation with Sulfo-NHS-SS-Biotin (Thermo Scientific; Waltham, MA, USA) at 4 °C for 30 min, according to the manufacturer’s instructions. Cells were lysed on ice for 10 min in lysis buffer containing a protease inhibitor cocktail (Roche, Rotkreuz, Switzerland) and were centrifuged at 10,000g at 4 °C for 2 min. Biotin-labeled surface proteins were captured on neutravidin agarose resin (Thermo Scientific) at room temperature for 1 h. The resin was washed five times with wash buffer containing a protease inhibitor. Proteins bound to the resin were eluted with SDS PAGE sample buffer and analyzed by Western blotting.

### 4.9. Chemicals and Statistical Analysis

Chemicals were of the highest purity possible and were from Sigma-Aldrich (Deisenhofen, Germany). Data are reported as means ± SEM. Student’s *t*-tests (for paired or unpaired samples as appropriate) or ANOVA were used for statistical analysis. A value of *p* < 0.05 was accepted as a significant difference. 

## 5. Conclusions

Taken together, the housekeeper protein ANO6 and other anoctamins have overlapping properties and are typically co-expressed [13]. These anoctamins scramble membrane PLs and may thereby affect intracellular trafficking and the PM expression of other ion channels, such as other anoctamins, CFTR or Orai1 (Supplement 1) [6,8,9,19,25,32,42]. Anoctamins change cellular Ca^2+^ signaling indirectly or even directly by operating as Ca^2+^ permeable channels [2,36]. Thus, a significant functional interdependence of anoctamins exists, which may impair the analysis of protein function, particularly in overexpression studies. Endogenous ANO6 adds low scrambling activity but otherwise does not seem to regulate scramblase activity by overexpressed scramblases. Although scramblase activity has been reported for ANO4 and ANO10 [43,44,45,46], no scrambling activity was detected for ANO4, -8 or -10 in KO-ANO6 cells in the present study (Figure 5). However, as only PM scrambling was assessed in the present study, our results do not exclude PL scrambling by ANO4, -8, or -10 in intracellular membranous compartments.

## Figures and Tables

**Table 1:** Primers used in this study.

Gene Accession Number	Primer	Size (bp)
human ANO1 NM_018043	s: 5′- CGACTACGTGTACATTTTCCG as: 5′- GATTCCGATGTCTTTGGCTC	445
human ANO2 NM_001278596	s: 5′- GTCTCAAGATGCCAGGTCCC as: 5′- CTGCCTCCTGCTTTGATCTC	553
human ANO3 NM_001313726	s: 5′- CTTCCCTCTTCCAGTCAAC as: 5′- AAACATGATATCGGGGCTTG	461
human ANO4 NM_001286615	s: 5′- CGGAAGATTTACAGGACACCC as: 5′- GATAACAGAGAGAATTCCAATGC	505
human ANO5 NM_213599	s: 5′- GAATGGGACCTGGTGGAC as: 5′- GAGTTTGTCCGAGCTTTTCG	713
human T ANO6 NM_001025356	s: 5′- GGAGTTTTGGAAGCGACGC as: 5′- GTATTTCTGGATTGGGTCTG	325
human ANO7 NM_001370694	s: 5′- CTCGGGAGTGACAACCAGG as: 5′- CAAAGTGGGCACATCTCGAAG	470
human ANO8 NM_020959	s: 5´- GGAGGACCAG CCAATCATC as: 5´- TCCATGTCATTGAGCCAG	705
human ANO9 NM_001012302	s: 5′- GCAGCCAGTTGATGAAATC as: 5′- GCTGCGTAGGTAGGAGTGC	472
human ANO10 NM_018075	s: 5′- GTGAAGAGGAAGGTGCAGG as: 5′- GCCACTGCGAAACTGAGAAG	769
human GAPDH NM_001289726	s: 5′-GTATTGGGCGCCTGGTCAC as: 5′-CTCCTGGAAGATGGTGATGG	200
Exon 5, ANO6	s: 5′- GTATTTGTAAAAGTACACGCACC as: 5′- GAGGATGAGCCCATTCTCTG	463 404
Exon5, ANO6, Sequencing	s: 5′- CAAATGGAGGAGGAGGAGGA as: 5′- GAGGATGAGCCCATTCTCTG	796
Sequencing Primer	s: 5′- CAAATGGAGGAGGAGGAGGA	

## Data Availability

The data are available from the corresponding author upon request.

## Supplementary Materials

The following supporting information can be downloaded at: https://www.mdpi.com/article/10.3390/ijms25189998/s1.
