# Supplementary material for: Functional Interdependence of Anoctamins May Influence Conclusions from Overexpression Studies
Source: Int J Mol Sci. 2024 Sep 17;25(18):9998. doi: 10.3390/ijms25189998 (PMC11432102; doi:10.3390/ijms25189998)
Supplement: Supplementary file 1 [file ijms-25-09998-s001.zip › ijms-3169154-supplementary.pdf]

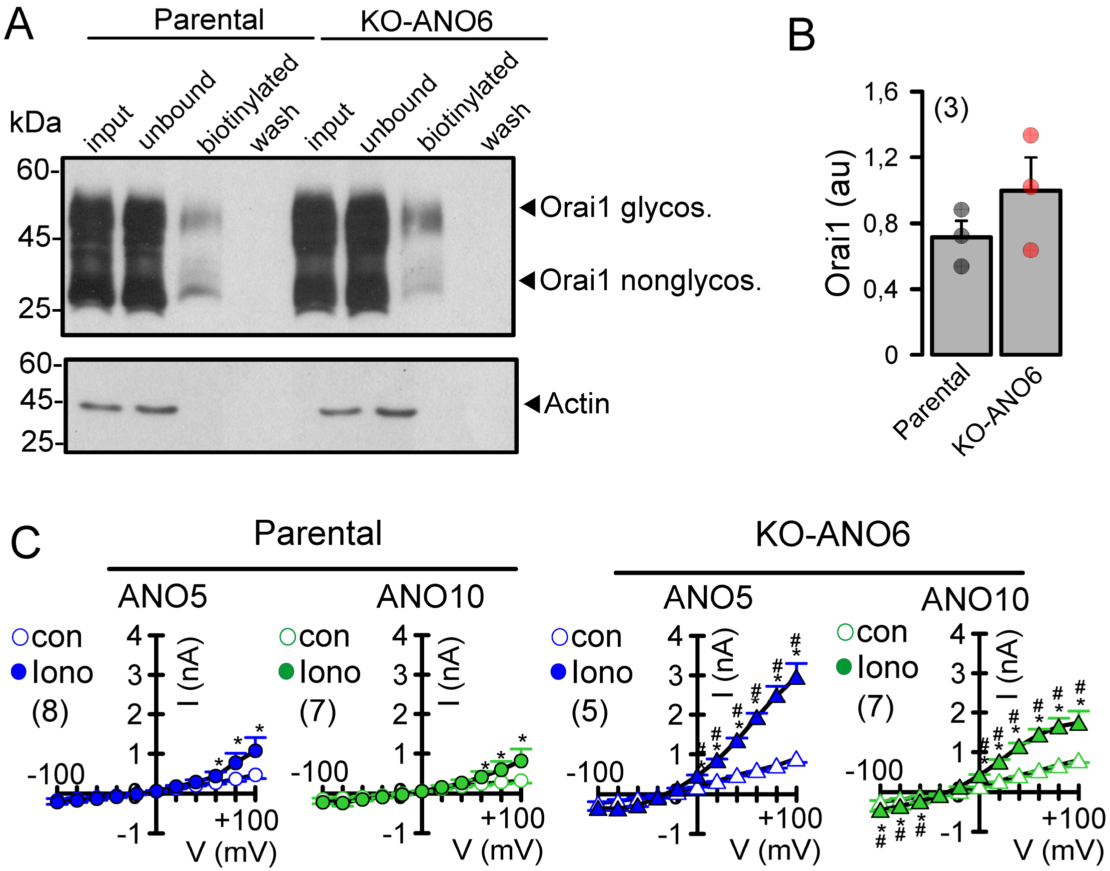

Supplementary Figure S1. *KO-ANO6 cells show augmented plasma membrane expression of Orai1 and increased activation of whole cell currents produced by ANO5 and ANO10.* **A**) Overexpression of Orai1 in parental and KO-ANO6 cells. A fraction of the overexpressed Orai1 could be biotinylated, i.e. was expressed in the plasma membrane (PM), while most of the overexpressed protein remained intracellularly. PM-expression of Orai1 appeared enhanced in KO-ANO6 cells. Bands for glycosylated and nonglycosylated Orai1 were detected. **B**) Summary of a semiquantitative analysis of Orai1-mRNA expression suggested no differences between parental and KO-ANO6 cells. **C**) Current/voltage relationships obtained in whole cell (wc) patch clamp experiments with parental and KO-ANO6 cells. Activation of wc currents by ionomycin (Iono; 1 µM) in cells expressing ANO5 or ANO10 was enhanced in KO-ANO6 cells, suggesting increased PM expression of ANO5 and ANO10, respectively, in KO-ANO6 cells. Mean ± SEM (number of experiments). *significant activation by Iono ($p = 0.012$ and 0.019). #significant difference when compared to parental ($p = 0.0091$ and 0.011; unpaired t-test).
